# Unleashing the power of shark variable single domains (VNARs): broadly neutralizing tools for combating SARS-CoV-2

**DOI:** 10.3389/fimmu.2023.1257042

**Published:** 2023-09-11

**Authors:** Olivia Cabanillas-Bernal, Blanca J. Valdovinos-Navarro, Karla E. Cervantes-Luevano, Noemi Sanchez-Campos, Alexei F. Licea-Navarro

**Affiliations:** Biomedical Innovation Department, Centro de Investigación Científica y Educación Superior de Ensenada, (CICESE), Ensenada, Baja California, Mexico

**Keywords:** VNAR, single-domain antibody, phage display, SARS-CoV-2, COVID-19

## Abstract

The pandemic caused by the severe acute respiratory syndrome coronavirus 2 (SARS-CoV-2) generated a joint global effort to develop vaccines and other treatments that could mitigate the negative effects and the rapid spread of the virus. Single-domain antibodies derived from various sources, including cartilaginous fish, camelids, and humans, have gained attention as promising therapeutic tools against coronavirus disease 2019. Shark-derived variable new antigen receptors (VNARs) have emerged as the smallest naturally occurring antigen-binding molecules. Here, we compile and review recent published studies on VNARs with the capacity to recognize and/or neutralize SARS-CoV-2. We found a close balance between the use of natural immune libraries and synthetic VNAR libraries for the screening against SARS-CoV-2, with phage display being the preferred display technology for the selection of VNARs against this virus. In addition, we discuss potential modifications and engineering strategies employed to improve the neutralization potential of VNARs, such as exploring fusion with the Fc domain of human Immunoglobulin G (IgG) to increase avidity and therapeutic potential. This research highlights the potential of VNARs as powerful molecular tools in the fight against infectious diseases.

## 1 Introduction

In December 2019, the Wuhan Municipal Health Commission in Wuhan City, China, reported to the World Health Organization the existence of hundreds of cases of atypical respiratory diseases. By January of 2020, a novel, highly transmissible coronavirus called severe acute respiratory syndrome coronavirus 2 (SARS-CoV-2), responsible for causing the coronavirus disease 2019 (COVID-19), was identified (1). SARS-CoV-2 is a single-stranded RNA virus, belonging to the family *Coronaviridae*, order *Nidovirales*, and is classified under the subfamily *Orthocoronavirinae* as the *betacoronavirus* genus (2, 3).

The pandemic caused by SARS-CoV-2 became a global health crisis and generated a joint worldwide effort to develop vaccines and other treatments for COVID-19 that could help stop the rapid spread of the virus. This resulted in several vaccines against COVID-19, being licensed for emergency use in the United States and other countries, including the Pfizer-BioNtech (BNT162b2) (4), Moderna (mRNA-1273) (5) nucleoside-modified messenger RNA–based vaccines, and the Johnson & Johnson (J&J)/Janssen (Ad26.COV2.S) (6). The high mutation rate exhibited by single-stranded RNA (ssRNA) viruses, such as SARS-CoV-2, leads to the continuous emergence of new variants that possess increased infectious potential. These variants have shown the ability to evade vaccine-induced immunity, thereby limiting the efficacy of current vaccines against this virus (7, 8). Consequently, it becomes imperative to explore alternative antiviral treatments. These treatments can be particularly valuable in situations where patients have not been vaccinated or when their immune systems exhibit a poor response to COVID-19 vaccines.

Two main therapeutic targets have played a pivotal role in addressing the pathogenesis of COVID-19: controlling the host’s cell response and managing the virus replication cycle (9). Initial efforts focused on controlling host-related cell responses, using convalescent plasma from recovered patients or administering corticosteroids and interferons; these approaches were approved as immunotherapies by the Food and Drug Administration (FDA) in March 2020. Simultaneously, efforts were made to target the virus itself by repurposing existing antiviral molecules and investigating new immunotherapies. Various stages of the virus’s life cycle, including entry, protease inhibition, anti-RNA polymerase activity, and release inhibition, were explored using molecules like umifenovir, nelfinavir, lopinavir-ritonavir, remdesivir, ribavirin, and oseltamivir, based on promising *in vitro* results (9–11). As of now, Remdesivir, an intravenously administered nucleotide prodrug, is the only FDA-approved antiviral drug for the treatment of COVID-19, whereas Ritonavir-boosted nirmatrelvir (Paxlovid) and molnupiravir have received Emergency Use Authorizations (EUA) from the FDA.

Neutralizing monoclonal antibodies (mAbs) have proven effective in targeting various proteins involved in the replication cycle of coronaviruses (12, 13). Nearly all mAbs developed for COVID-19 specifically target the SARS-CoV-2 spike protein (14, 15), which binds to the host cell mediating entry of the virus. By blocking viral attachment and entry into human cells, these mAbs provide a potential solution. However, it is worth noting that mAbs directed against the receptor-binding domain (RBD) of the spike protein exhibit reduced efficacy against certain variants (16). To address this challenge, combinations of human mAbs have been found to enhance neutralizing activity against variants of concern with antigenic substitutions in the RBD (17, 18). According to the NIH COVID-19 treatment guidelines, only four anti–SARS-CoV-2 mAb products (bamlanivimab/etesevimab, casirivimab/imdevimab, sotrovimab, and bebtelovimab) have received EUA from the FDA for the treatment of mild to moderate COVID-19. However, because of the emergence of coronavirus subvariants that exhibit resistance to mAbs, their efficacy in prevention and treatment is expected to be limited (19).

Conventional human IgG (hIgG) antibodies are composed of two heavy and two light chains (20). Their complex structure and large size of approximate 150 kDa have limited their use in certain applications such as intracellular targeting or oral administration. In recent years, advances in the structure and function of antibodies have led to the development of different antibody formats that seek to reduce the size of conventional IgG to minimal antibody fragments, resulting in the development of single-domain antibodies (sdAbs) with only a variable regions of heavy (VH) or a light (VL) domain, that retain parental antigen binding. As an alternative to engineered human sdAbs, two unique antibody isotypes have been found naturally containing a single domain for antigen recognition. This includes the heavy-chain antibodies found in the blood serum of Camelidae that have the variable domains of heavy-chain-only antibodies (VHH) or nanobodies sdAbs (21) and the new antigen receptor from cartilaginous fish that possess sdAbs named as VNAR (22).

The VNARs were first reported by Greenberg et al. (22) for the immune repertoire of the nurse shark (*Ginglymostoma cirratum*). VNARs possess a unique arrangement of the complementarity-determining regions (CDRs), with the presence of the CDR1 and CDR3 loops, lacking the CDR2 loop present in the rest of the known antigen-binding domains. In absence of a CDR2 loop, VNARs possess two additional hypervariable loops with elevated rate of somatic mutations, namely, hypervariable region 2 (HV2) and HV4, also involved in antigen recognition (23, 24). VNARs present special characteristics such as a small size of approximately 12 kDa, being the smallest antigen-binding domains naturally found, and a long and extended CDR3, which can recognize protein motifs inaccessible to classical antibodies (25–27). VNARs have shown other advantages over conventional antibody molecules such as high thermal and chemical stability as well as good tissue penetration (28–33). Their special and unique properties are desirable for the development of new drugs and makes them ideal candidates as potential therapeutic agents for a wide range of applications, including cancer treatment, imaging, drug delivery, and antiviral applications, where neutralizing molecules are needed that can recognize cryptic epitopes inaccessible to conventional human antibodies that are impervious to mutational drift (34). In the present work, we compile and review recent published studies on VNARs with the capacity to recognize and/or neutralize SARS-CoV-2. We found that shark-derived single-domain VNARs are broadly neutralizing agents for different variants of the SARS-CoV-2, including Wuhan strain and Alpha, Beta, Delta, and Omicron variants. The works on this topic suggest that VNARs can be used in different formats such as monomers or fused to the crystallable fraction (Fc) of hIgG as monovalent or divalent fusion protein to develop antibody-based drugs against current variant of concern (VoC) and future variants.

## 2 Broadly neutralizing shark-derived single-domain antibodies to SARS-CoV-2

Using phage display technology and biopanning selection, Gauhar et al. (35) conducted a screening against the S1-RBD domain and S1 subunit of SARS-CoV-2 spike protein, using two VNAR semi-synthetic phage libraries, OSX3 and OSX6 (36), that were constructed on the basis of type II VNAR nurse shark sdAb sequences. From the last two rounds of selection, 149 unique VNARs were identified to recognize any of the antigens used (94 for S1-RBD and 55 for S1). By cloning into a pFUSE expression vector, the 149 unique VNARs were reformatted into bivalent hIgG Fc-fusion (VNAR-hFc). From the reformatted VNAR-hFc, 56 clones showed high affinity and specificity to S1-RBD and/or S1 protein from the Wuhan variant. Nine of these 56 unique clones showed binding only to S1. Ten VNAR-hFc clones were also tested against two key mutations in the S1-RBD region, N501Y and E484K, found in newly emerged SARS-CoV-2 variants at that time. Nine of the 10 clones tested maintained binding to S1-RBD N501Y comparable with that of S1-RBD of the original Wuhan variant. For S1-RBD E484K, binding was reduced for all clones tested compared with S1-RBD of the original Wuhan variant. These 10 clones were further studied, because of their ability to block the *in vitro* binding of the recombinant angiotensin-converting enzyme 2 (ACE2) receptor to S1 and S1-RBD from the Wuhan variant as well as the S1-RBD N501Y mutant, in a competition Enzyme-linked immunosorbent assay (ELISA). All 10 clones neutralized live Wuhan strain in *in vitro* cell infectivity assays, using Vero CCL81 cells. The authors conclude that screening and selection of clones using *in vitro* and cell-based assays could accurately predict the inhibition potential when using live virus and propose VNARs against the spike protein of SARS-CoV-2, as novel therapeutic approaches against COVID-19. This work resulted in the patent US11345742 (37), the only patent granted to date of VNARs to treat COVID-19.

Ubah et al. (34) isolated a series of VNAR from a synthetic VNAR phage display libraries using a biopanning selection against the SARS-CoV-2 RBD. After four rounds of selection, 90 individual clones from each round were used to obtain populations of monoclonal VNAR-presenting phage and the antigen binding was assessed using an ELISA. VNARs that bound to RBD in ELISA were tested for neutralization of SARS-CoV-2 and SARS-CoV-1 pseudoviruses, using ACE2-expressing human embryonic kidney cells (HEK293T cells) and a luciferase assay. From this screen, three VNARs (3B4, 2C02, and 4C10) with half-maximal inhibitory concentration (IC_50_) values < 10 nM were selected for further characterization. The three selected VNARs were able to neutralize pseudovirus SAR-CoV-2 and SARS-CoV-1 *in vitro*, as well as the pseudovirus WIV1-CoV, a pre-emergent zoonotic virus. Only VNAR-3B4 was able to neutralize pseudotype with Middle East respiratory syndrome coronavirus (MERS-CoV) spike proteins, maybe due to small size and protruding CDR3 that makes it capable to access and bind to an interface that is conserved among beta-coronavirus lineages (class 2B and class 2C). The three VNARs evaluated were able to neutralize live SARS-CoV-2 (strain USA_WA1/2020) *in vitro* in Vero E6 cells. In this assay with the live virus, 2C02 and 4C10 were shown to be more potent, whereas 3B4 had a slight loss of potency, compared with data collected from pseudovirus experiments. VNARs 3B4 and 2C02 were furtherly studied by crystallographic analysis in complex with SARS-CoV-2 spike RBD, and results showed that the two VNARs bind to distinct and on opposite sides of the RBD and that neither of them overlaps with the ACE2 receptor interface. The crystal structures also suggested that VNAR-3B4 only recognizes and binds to an epitope on RBD when it is in the “up” conformation, whereas VNAR-2C02 is capable of binding to an epitope available in both “up” and “down” conformations of the RBD. By mapping reported mutations sites on the RBD, the authors found that most of the RBD mutations were distant from the VNAR-3B4 binding site but directly at the VNAR-2C02 epitope, suggesting that VNAR-3B4 might maintain its neutralizing activity against several SARS-CoV-2 variants, whereas VNAR-2C02 could lose the ability to bind and neutralize some variants that include the mapped mutation sites. Using a biolayer interferometry (BLI), it was confirmed that the mutations found in the Beta variant (also found in other variants of concern at those dates) had little or no effect on the binding affinity of 3B4. Finally, this work proposes VNARs as neutralizing agents of different variants of the SARS-CoV-2 virus and, on the basis of their different recognition sites, proposes that 3B4 and 2C02 could be a potential therapeutic, for use as a cocktail of antibodies.

Through the immunization of a bamboo shark (*Chiloscyllium plagiosum*) with SARS-CoV-2 S protein, Feng et al. (38) constructed and screened an immune VNAR phage library. After three rounds of biopanning, 26 unique RBD-specific “vnarbodies” were obtained, clones 20G6 and 17F6 being the ones that presented the highest half-maximal effective concentration (EC_50_). The 20G6 and 17F6 were expressed as VNAR monomers and were subsequently reformatted and expressed as dimer IgG1 Fc-fusion proteins by cloning into pCMV3-IgG1 vector. The divalent 20G6-Fc and 17F6-Fc VNARs were found to bind to RBDs of several SARS-CoV-2 variants including RBDs of Alpha, Beta, Kappa, Delta, and Delta plus and Lambda variants. Using a pseudotyped virus neutralization assay, it was found that monovalent and divalent 20G6 and 17F6 VNARs can neutralize SARS-CoV-2 Wuhan, Beta, Delta, Delta plus, Kappa, and Lambda variants with IC_50_ at the nanomolar range. In addition, using a focus reduction neutralization test 50 assay, all monovalent and divalent 20G6 and 17F6 VNARs showed ability to neutralize authentic SARS-CoV-2 Wuhan strain as well as Beta and Delta variants, being the divalent formats, those that presented a higher neutralization efficiency (2–10 times more) compared with its parental VNAR monomer. This work evaluates for the first time the *in vivo* prophylactic and therapeutic potential of a VNAR (20G6-Fc) in two different mouse infection models (SARS-CoV-2 Wuhan and Beta variant). For the prophylactic evaluation, the 20G6-Fc was intranasally administrated to hACE2-transgenic C57BL/6 mice, 3 h before the infection with SARS-CoV-2 Wuhan. On day 3 after infection, the genomic RNA viral load in the lungs was found to be reduced by 1.5 logs in the 20G6-Fc–treated compared with the untreated control group. The prophylactic potential of 20G6-Fc against SARS-CoV-2 Beta variant was evaluated in BALB/c mice, because this variant can infect wild-type mice. The 20G6-Fc was intranasally administrated to BALB/c mice, 3 h before the challenge with SARS- CoV-2 Beta variant. On day 3 after infection, the genomic RNA viral load in the lung was found to be reduced by 4.5 logs in the 20G6-Fc–treated mice compared with the control group treated with an unrelated VNAR. For the therapeutic potential evaluation of 20G6-Fc in mice, hACE2-transgenic C57BL/6 mice intranasal challenge with SARS-CoV-2 Wuhan were treated with 20G6-Fc administrated via intraperitoneal injection at 3 h after challenge, whereas BALB/c mice infected with SARS-CoV-2 Beta strain were treated with 20G6-Fc administrated via intranasal instillation at 3 h after challenge. On day 3 after infection, the genomic RNA viral load in the lung of 20G6-Fc–treated mice was found to be reduced 2.2 logs for the mice intranasal challenge with SARS-CoV-2 Wuhan, whereas RNA viral load was found to be reduced 2.7 logs for the mice infected with SARS-CoV-2 Beta strain. Histopathological analysis of the lung showed a significant reduction in lung pathology in the 20G6-Fc–treated mice for the prophylactic and therapeutic evaluation. Both 20G6 and 17F6 VNARs were furtherly studied by crystallographic analysis in complex with SARS-CoV-2 spike RBD (N501Y). The unveiled crystal structures of 20G6 complexed with RBD indicated that it binds to a conserved epitope region (365–380) on the RBD, outside of ACE2 binding site. Of note, an atypical interaction mechanism was observed as the beta-strand of CDR3 of 20G6 interacts with RBD that is mainly supported by hydrophobic interactions. Furthermore, the side chain of arginine distributed among the CDR3 can form additional hydrogen bonds with RBD, enhancing the affinity of 20G6. In this work, Feng et al. demonstrate that intranasal administration of VNAR-Fc fusion could be a promising tool for protection against SARS-CoV-2 Wuhan and Beta variant in both prophylactic and therapeutic models.

According to the work by Chen et al. (39), a VNAR phage library from *C. plagiosum* sharks immunized with SARS-CoV-2 RBD was panned using phage display technology. Four unique VNARs with distinct CDR3 were identified to target SARS-CoV-2 RBD. It was shown that the four VNARs studied were highly thermostable, presenting melting temperatures (T_m)_ from 54.38°C to 56.39°C, using a thermal shift assay. The four unique VNARs were reformatted into a hIgG-like molecule by fusing the VNAR to a hIgG1 Fc in a mammalian expression vector (VNAR-Fc). The binding affinity of the Fc-Free VNARs and the VNAR-Fc fusion was also evaluated using BLI, and it was found that the VNAR-Fc fusion increased RBD binding affinity (with K_D_ values of 28.3 nM, 3.88 nM, 211 nM, and 9.20 nM for JM-2-Fc, JM-5-Fc, JM-17-Fc, and JM-18-Fc, respectively), compared with its parental VNAR monomer (429 nM, 38.5 nM, 2720 nM, and 60.3 nM for JM-2, JM-5, JM-17, and JM-18, respectively). The EC_50_ values of the VNAR-Fc fusion determined by ELISA confirmed that JM-5-Fc and JM-18-Fc fusions had the strongest RBD binding (with EC_50_ values of 0.190 nM and 1.437 nM, respectively). The binding to the RBD of the Delta and Omicron variants was also evaluated by ELISA, finding that the four VNAR-Fc fusions maintained their ability to bind to the RBD of the Delta variant, whereas only JM-5 retain strong binding to the omicron RBD. This work also evaluated the capacity of the VNAR-Fc fusion in blocking RBD-ACE2 interaction, finding that the four VNAR-Fc fusions were capable of blocking the interaction ACE2-WT RBD and ACE2-WT RBD, with JM-2-Fc being the one that showed the best blocking capacity. None of the VNAR-Fc fusions showed obvious blocking of the ACE2-Omicron RBD interaction. The epitope competition of the VNAR-Fc fusions was analyzed by a BLI, and it was found that only two of the four VNARs (JM-5 and JM-17) showed epitope competition. On the basis of this epitope competition results, five bi-paratopic VNAR (Fc-free) constructs with non-overlapping epitopes were designed, and their affinity was studied by BLI. Four bi-paratopic VNARs showed an enhanced binding activity, compared with the monovalent VNARs. The binding affinity was further increased by fusing bi-paratopic VNARs to Fc. Using an ELISA, it was confirmed that the five bi-paratopic VNAR-Fc fusions increased their binding to the RBDs of WT SARS-CoV-2, Delta and Omicron variants, and SARS-CoV, compared with the VNAR monomers. This is the first report that studies and proposes biparatopic VNAR formats against SARS-CoV-2 as diagnostic and therapeutic agents for COVID-19.

Using phage display technology, Valdovinos-Navarro et al. (40) isolated a novel VNAR aimed at the SARS-CoV-2 spike protein by screening three previously reported synthetic VNAR phage display libraries, from the *Heterodontus francisci* shark (41). A few modifications were conducted during the biopanning (42) as plasma from patients with convalescent COVID-19 was used for phage elution. After four rounds of biopanning, 72 clones were selected, and four unique VNAR sequences were identified. The binding activity of VNARs to the SARS-CoV-2 RBD was assessed by ELISA, and the VNAR SP240 was identified as the best binder. The neutralizing activity of VNAR SP240 was *in vitro* evaluated against the Delta (B.1.167.2) and Omicron (B.1.1.529) variants using lung epithelial cell line A549-hACE2-TMPRSS2 and Vero E6 cell line. In the A549-hACE2-TMPRSS2, the VNAR SP240 displayed NT50 of 0.1833 μg/mL (12.06 nM) against Omicron and NT50 of 0.8818 μg/mL (58.01 nM) against the Delta variant. Furthermore, the inhibition rate of VNAR SP240 was higher than 90% for both SARS-CoV-2 variants. In Vero E6 cells, higher NT50 values of VNAR SP240 were obtained: 4.622 μg/mL (304 nM) for Omicron and 6.737 μg/mL (442.4 nM) for Delta. Hence, higher amounts of VNAR SP240 were required to block the SARS-CoV-2 infection in more than 90% (10 μg/mL). By *in silico* modeling, it was revealed that the CDR3 of VNAR SP240 plays a predominant role during the binding of the spike protein. The CDR3 loop directly engages the receptor-binding motif in the spike protein of both variants, suggesting that the neutralizing mechanism occurs through direct blocking of the binding surface to the ACE2 cellular receptor. Moreover, collaboration from other variable loops like HV2 and CDR1 for antigen binding was observed for the VNAR SP240. The sequence variability among SARS-CoV-2 variants, particularly in the spike protein, seemed not to decrease either the SP240 activity or the binding energy to its antigen, suggesting a potential reactivity of SP240 against a wide spectrum of variants. Therefore, this work highlights the potential as an ideal molecular tool to develop antibody-based drugs against future variants.

In a very elegant work, Chen et al. (43) isolated a series of VNARs from nurse sharks immunized individually with SARS-CoV-2 recombinant RBD, RBD-ferritin (RFN), or spike protein ferritin nanoparticle (SpFN). Each antigen showed a different immune response of antibodies against SARS-CoV-2 after three or four immunizations. Immune VNAR phage display libraries containing >10^10^ members in size were built for each antigen. The VNAR libraries were panned against its target antigen. By cloning into a mammalian expression vector upstream of the hIgG1 Fc encoding sequence, the selected unique antigen-positive VNARs were reformatted as hIgG1 Fc-fusion chimeras (ShAbs). The chimera molecules were evaluated by ELISA to recognize SARS-CoV-2 RBD and S-2P, and all ShAbs showed recognition by both target immunogens. Two ShAbs (ShAb01 and ShAb02) isolated from RBD-immunized sharks were further studied, showing robust binding to both SARS-CoV-2 RBD and S-2P with K_D_ values in the nanomolar range. Both ShAbs showed binding with affinity in the nanomolar range to SARS-CoV-2 RBD WA-1, Alpha, Beta, Gamma, Delta, and some Omicron variants. The ability of the ShAb01 and ShAb02 chimeras to neutralize a series of SARS-CoV-2 pseudoviruses was also evaluated. From this study, ShAb01 showed strong neutralization against SARS-CoV-2 WA-1, Alpha, Beta, and Delta variants and the heterologous SARS-CoV-1, with IC_50_ of 188–873 ng/mL, whereas ShAb02 neutralized SARS- CoV-2 WA-1, Alpha, Beta, and Delta variants, with IC_50_ values of 15–52 ng/mL, showing reduced neutralization against SARS-CoV-1 (11.6 μg/mL). This work also evaluates the protection ability of an anti–SARS-CoV-2 VNAR in an *in vivo* model. K18- hACE2 transgenic mice, expressing human ACE2 on their epithelial cells, were passively immunized with ShAb01, ShAb02, or an IgG1 isotype control; after 24 h, mice were subjected to an intranasal challenge with SARS-CoV-2 WA-1/2020. After 14 days of monitoring, both ShAbs tested provided significant protection compared with control IgG1, with ShAb01 being the one that provided the greatest protection with late onset of the disease with a survival rate of 86% (compared with 43% for ShAb02 group and 0% for the control group). This work also performed structural studies of the ShAbs with SARS-CoV-2, finding that ShAb01 and ShAb02 bind to epitopes on opposite sides of the RBD, in a sandwich-like complex. On the basis of their distal epitopes, multidomain molecules were designed, containing ShAb01, ShAb02, or both VNARs. The results suggested that two bispecific engineered molecules (ShAb01H02K and BiShAb0201) showed large improvements in antigen-affinity, with >10-fold increased affinity for all SARS-CoV-2 VoC RBDs over ShAb01 or >3-fold increased affinity over ShAb02. The ability of the bispecific molecules to neutralize SARS-CoV-2 pseudoviruses compared with parental VNARs was evaluated, finding that ShAb01H02K and BiShAb0201 increased their neutralization levels >18–667 fold compared with ShAb01 and >28 fold compared with ShAb02. This work suggests that the multimerization of the VNAR domains or their fusion to an Fc fraction could increase the therapeutic half-life of these domains, and based on the fact that the different VNARS formats evaluated that included VNARs by themselves, Fc-fused formats or multispecific molecules have the ability to neutralize a SARS-CoV-2 VoC panel, Chen et al. propose VNARs as an alternative strategy to conventional neutralizing antibody tools, which, taking advantage of its characteristics such as small size, could be used in easy-to-administer therapeutic formulations, such as aerosolization.

According to the work by Buffington et al. (44), a previously reported naïve nurse shark VNAR phage display library (45) was screened against the SARS-CoV-2 wild-type S2 subunit. After three rounds of panning, 53 unique S2-binding VNARs were identified. In addition, sequencing phage pools from selection rounds 2 and 3, VNAR S3B3 was identified as the most significantly enriched clone, despite that this S3B3 was not one of the clones with the best neutralization activity, as will be noted later. All unique VNAR clones were tested in ELISA against S2 and full-length ectodomain spike trimer. The best candidates from this assay were tested for neutralization of wild-type SARS-CoV-2 pseudoviruses, using ACE2-expressing 293T cells. Four VNARs—S2A9, S2G8, S3A10, and S4A9—were tested for binding activity against the S2 subunit of other β coronaviruses, and all four binders were found to have cross-activity against the S2 subunit of MERS, SARS-1, and HCoV-43. Because it presented the best neutralization activity and the best binding to different S2 subunits with best binding activity to SARS-CoV-2 S2, S2A9 VNAR was selected for further characterization. The binding affinity of S2A9 was calculated by mass photometry (MP) with K_D_ values of 590 nM. Sandwich ELISA was carried out to predict the binding epitope of S2A9, using the human anti-S2 antibody COV44-62 and the commercial mouse antibody 1A9 anti-S2. The results suggested that the S2A9 and 1A9 epitopes in the S2 subunit might overlap, whereas the data from S2A9 and COV44-64 were inconclusive. The VNAR S2A9 was reformatted into bivalent hIgG Fc-fusion (S2A9-hFc). In both the parental VNAR monomer and bivalent Fc-fusion protein, S2A9 was able to neutralize wild-type pseudotyped SARS-CoV-2 as well as the VOCs: Alpha, Beta, Delta, Gamma, Lambda, and Omicron subvariants, with the S2A9-hFc format being the one that presented the highest neutralization efficiency, up to 36 times higher than its monomer version in the wild-type pseudovirus. Finally, S2A9 VNAR also showed neutralization ability against live ancestral SARS-CoV-2 strain WA (IC_50_, 3,601 nM) and all VOCs tested, whereas the S2A9-hFc format was not able to neutralize any of the tested variants including original virus in the neutralization assays with live virus. This work proposes VNARs as potential tools to neutralize emerging variants of SARS-CoV-2 and highlights the potential of naive phage libraries for the rapid screening of antibodies against viral pathogens.

In the most recent work on anti–SARS-CoV-2 VNARs, Kim et al. (46) report for the first time the immunization of banded houndshark (*Triakis scyllium*) for the isolation of VNAR-based therapeutics. Recombinant SARS-CoV-2 wild-type RBD protein was used to immunize the shark and construct a phage display immune library, which was then panned against the RBD as the target antigen during three rounds of selection. From the biopanning, 33 RBD-specific clones were isolated, 31 were identical in sequence (CoV_2_NAR-1), whereas the other two (CoV_2_NAR-2 and CoV_2_NAR-3) presented unique sequences. The EC_50_ values determined by ELISA for the three VNARs were 1.6 nM, 5.8 nM, and 4.5 nM, for CoV_2_NAR-1, CoV_2_NAR-2, and CoV_2_NAR-3, respectively, with CoV_2_NAR-1 being the one that presented the highest binding affinity for the RBD. CoV_2_NAR-1 was further studied, showing robust binding affinities to RBDs of SARS-CoV-2 Alpha, Beta, and Delta VoC. A high thermal stability of CoV_2_NAR-1 was demonstrated, maintaining its binding affinity after incubation at 80°C for 1 h. The ability of CoV_2_NAR-1 to neutralize wild-type SARS-CoV-2 pseudovirus was also evaluated, showing a strong neutralization with an IC_50_ of 660 nM. The CoV_2_NAR-1 was reformatted into bivalent version by fusing to hIgG1 Fc CH3 domain, and, as expected, the IC_50_ of the bivalent CoV_2_NAR-1 was considerably improved by two orders of magnitude (up to a hundred times) over the parental monovalent VNAR, showing a broad neutralization activity in the nanomolar range against the wild-type SARS-CoV-2 as well as Alpha and Delta variants, using a pseudotyped virus neutralization assay. The work of Kim et al. highlights the potential of the immune repertoire of the banded houndshark (previously reported only for the construction of semi-synthetic libraries) as an attractive source of VNAR-based therapeutics or diagnostics against pathogens of interest.

## 3 Conclusions and prospects

sdAbs derived from different sources such as cartilaginous fish (30, 47, 48), camelids (49–51), or humans (52, 53) have emerged as promising molecular tools for antigen recognition and neutralization, due to their small size, up to 10 times smaller than a conventional IgG antibody. Shark-derived VNARs are known as the smallest naturally occurring antigen-binding molecules, with molecular weights ranging from 12 to 15 kDa. After all their proven advantages, shark antibodies have been understudied compared with their camelid counterpart, although the discovery of both occurred only a few years apart (21, 22). This can be clearly seen in the number of publications to date on camelid-derived antibodies against SARS-CoV-2, compared with the publications on isolated VNARs against this virus. This greater inclination toward the study of camelid nanobodies is attributed to the limited availability of shark models, due to the difficulty of maintaining most reported shark species for VNAR isolation, in captivity (because of their large size and aggressive behavior) (46, 54).

**Figure 1:**
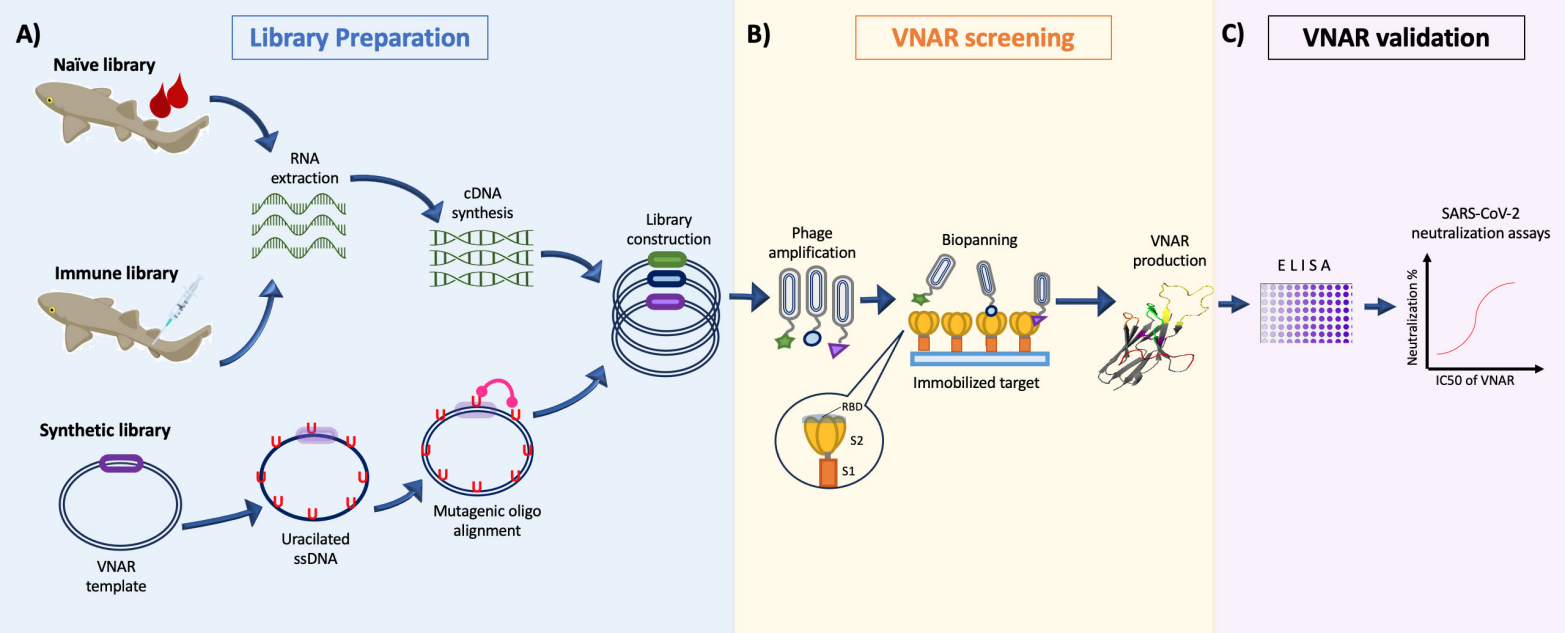
General representation of the isolation process of VNARs against SARS-CoV-2. **(A)** Construction of the phage-displayed Naïve library from unimmunized sharks (Buffington et al., 44); immune library immunizing sharks multiple times with SARS-CoV-2 proteins (Chen et al., 43); or synthetic libraries diversifying the CDR3 loop with mutagenic oligonucleotides (Cabanillas-Bernal et al., 41). **(B)** Screening of VNAR clones by Biopanning using the S1, S2, or RBD domain of the Spike protein as antigen during selection. **(C)** The candidate clones are evaluated in a *in vitro* virus neutralization assay to corroborate their SARS-CoV-2 neutralizing activity.

It has been reported that VNARs can be isolated from libraries of immune (30, 47, 48), naïve (45, 55), synthetic (41), or semi-synthetic origin (56) (**Figure 1**). The diversity of the VNAR libraries screened in the reviewed works was 50% from natural immune sources (38, 39, 43, 46), 37.5% from synthetic origin (34, 35, 40), and 12.5% from natural naïve (non-immunized) source (44). Whereas the immune libraries screened in the reviewed papers were constructed using similar protocols for immunization and amplification of the shark immune repertoire, the synthetic libraries screened were constructed by different methodologies. Gauhar et al. (35), used type 2 nurse shark VNAR semi-synthetic libraries generated by overlap PCR, with variable CDR3 lengths, incorporating randomization of this region by NNK codons (36). In their work, Ubah et al. (34) screened a synthetic VNAR library, constructed by combining naïve VNAR frameworks, different CDR3 lengths, generating diversity within the CDR1 and CDR3 and incorporating some non-canonical cysteine residues into CDR1 and CDR3. Valdovinos-Navarro et al. (40) screened three previously reported horn shark VNAR synthetic libraries (41) constructed by Kunkel Mutagenesis, each with a different CDR3 length, as well as different numbers of cysteines in this region, including sequence diversity only within the CDR3 by NNK codons. When new pathogens emerge and spread in an unexpected and very fast way as in the case of SARS-CoV-2, synthetic antibody libraries can offer a fast and reliable source for the search for neutralizing antibodies because the selection of sdAbs from a synthetic library requires 2–3 weeks, whereas generation of sdAbs from immunized libraries needs at least 4 months from animal immunization to antibody selection. This is highlighted by the fact that the first studies on anti–SARS-CoV-2 VNARs used synthetic libraries for screening. Even so, many works tend to continue betting on the safe side, with the construction and screening of immune libraries, specific for the target antigen. In addition to the use of immune and synthetic libraries, Buffington et al. (44) used a naïve VNAR library constructed from peripheral blood leukocytes from six non-immunized adult nurse sharks (45). Buffington et al. propose the naïve shark VNAR phage display libraries as a promising platform for the rapid isolation of sdAbs with therapeutic potential.

Several antibody surface display technologies exist, including ribosome display, yeast surface display, bacterial surface display, and phage display. It has been reported that camelid sdAbs against SARS-CoV-2 have been isolated using display technologies other than phage display. Schoof et al. (57) screened a yeast surface-displayed synthetic library of llama nanobodies, against a mutant form of SARS-CoV-2 Spike (Spike^S2P^), identifying nanobodies capable of neutralizing SARS-CoV-2 pseudotyped and live SARS-CoV-2 viruses. Nieto et al. (58) identified an alpaca nanobody specific for the RBD of the SARS‐CoV‐2 spike protein by screening an *E. coli* surface display immune library of alpaca nanobodies. The isolated nanobody was capable of neutralizes SARS‐CoV‐2 wild type and the D614G variant. Phage display technology was the unanimously preferred screening technology for the selection of VNARs against SARS-CoV-2 (**Table 1**); however, other works on the isolation of VNARs, not related to SARS-CoV-2, have used other protein presentation techniques other than phage display. Zielonka et al. (59) used yeast surface display to isolate high-affinity VNAR domains from the bamboo shark (*C. plagiosum*) VNAR repertoire, against three different antigens: EpCAM, HTRA1, and EphA2. Grzeschik et al. (60) developed a modified yeast display approach for screening of VNAR fused to the a-agglutinin protein Aga2p and a gene encoding TurboGFP that can be detected via GFP expression. This approach can yield high-affinity binders against a variety of therapeutically target as a convenient and cost-efficient alternative.

**Table 1:** SARS-CoV-2 neutralizing VNARs.

Reference	Library Origin	Shark	Display technology	VNAR format	Antigen for selection	Live SARS-CoV-2 Variants tested	VNAR name	Binding affinity (EC_50_)	IC_50_ [M]	K_D_
(35)	Semi-synthetic	Nurse shark (*G. cirratum*)	Phage display	Bi-paratopic	S1 subunit of SARS-CoV-2 spike protein (residues 16–685) and S1-RBD domain (residues 319–541)	Wuhan strain	6ID10_5, 6ID10_6, 3ID10_16, 3ID10_40, 6ID10_70, 6ID10_71, 6ID10_75, 3ID10_96, 3ID10_99, 6ID10_113	< 10 nM	For S1 subunit, from 1.0E-07 to 7.3E-08; and for S1-RBD, from 1.5E-07 to 8.9E-08	N/S
(34)	Synthetic	N/S	Phage display	Single-domain	Recombinant SARS-CoV-2 RBD	USA_WA1/2020	3B4	N/S	1.15E-08 ± 4.4E-09	17.2-60.3 nM
							2C02		8.39E-10 ± 1.5E-10	
							4C10		6.13E-10 ± 2.56E-10	
							2D01		4.60E-6 ± 1.2E-6	
(38)	Immune	Bamboo shark (*C. plagiosum*)	Phage display	Bi-paratopic	S1 protein of SARS-CoV-2 Wuhan	Wuhan strain; Beta and Delta variants	20G6	0.003 μg/mL	10.47E-09 ± 1.0E-09	< 10 pM
							17F6	0.007 μg/mL	29.35E-09 ± 6.70E-09	
(39)	Immune	Whitespotted bamboo shark (*C. plagiosum*)	Phage display	Bi-paratopic	Recombinant SARS-CoV-2 RBD (residues 321–591)	NA	JM-2	2.938 + 0.418 nM	N/S	429 nM
							JM-5	0.221 + 0.041 nM		38.5 nM
							JM-17	2.016 + 0.439 nM		2720 nM
							JM-18	0.343 + 0.325 nM		60.3 nM
(40)	Synthetic	Horn shark (*H. francisci*)	Phage display	Single-domain	Recombinant RBD protein	Delta and Omicron	SP240	N/S	5.8E-08 (Delta), 1.206E-8 (Omicron)	N/S
(43)	Immune	Nurse sharks (*G. cirratum*)	Phage display	Bi-paratopic	SARS-CoV-2 RBD, RBD-ferritin (RFN), spike protein ferritin nanoparticle (SpFN)	USA_WA-1/2020	ShAb01	N/S	4.29E-09 + 1.77E-09	14.9 nM
							ShAb02	N/S	3.93E-09 + 4.79E-09	85.7 nM
(44)	Naive	Nurse sharks (*G. cirratum*)	Phage display	Single-domain	S2 subunit of SARS-CoV-2 spike protein	Wuhan strain, 614G, Alpha, Beta, Delta, Gamma, Lambda, and Omicron (BA.1 and BA.1.1)	S2A9	363 nM	10.71E-07 ± 10.97E-07	590 nM
(46)	Immune	banded houndshark (*T. scyllium*)	Phage display	Bi-paratopic	Recombinant SARS-CoV-2 wild-type RBD protein	NA	CoV_2_NAR-1	1.6 nM (WT SARS-COV-2), 2.3 nM (Alpha), 290 nM (Beta), and 92 nM (Delta)	N/S	N/S

NA, not applicable; N/S, not specified.

More than half of published works about VNARs neutralizing SARS-CoV-2 came up with the idea of fusing these molecules to an Fc domain of a hIgG, in order to increase its avidity and neutralizing potential. Chen et al. (43) propose the use of a bi-paratopic VNAR-Fc fusion molecule. This strategy seems to increase the VNARs potential 10 times over VNAR monomers (38) and its therapeutic half-life time. However, this would neglect one of the main advantages of VNARs over conventional antibodies, the small size of 12 kDa. In addition, as VNARs show different neutralizing spectra in accordance with the location of the recognition sites of VNARs in the RBD molecule, Ubah et al. (34) propose the use of VNARs as cocktails with a combination of two or more VNARs. Using VNARs in a multi-neutralizing antibodies cocktail represents an option to target and neutralize by binding to nearby epitopes and retaining the small molecular size of VNARs. Whereas some works propose the fusion of VNARs to Fc domains, others are aiming to further reduce the size of neutralizing molecules. Dueñas et al. (61) aimed to bind the CDR3 of a VNAR with a small conotoxin backbone rich in disulfide bonds to produce non-natural antibodies (NaNoBodies). The NaNoBodies resulted in a molecule four times smaller the size of a VNAR, which retained the recognition for the antigen presented by the parental VNAR. This reformatting option represents an innovative example of applications of the VNARs and a breakthrough to reduce its immunogenicity or increase its penetration into tissues.

The COVID-19 pandemic generated an opportunity for VNARs to showcase their broad neutralizing abilities against a highly pathogenic virus and its ever-growing variants. Consequently, the VNARs are now considered a tool to counteract SARS-CoV-2. In addition, the pandemic lent research groups to move forward in novel generation platforms of the VNARs, improve its half-life time, reduce its size, and enhance its specificity and affinity. Efforts to understand the interaction dynamic have also been made, and they have found that VNARs recognize separate epitopes on the RBD and had unique neutralization mechanisms for the virus. The degree of hydrophobic pairing between the RBD and ACE2 has been described as very strong (62). In accordance with this, the high abundance of hydrophobic side chains on the binding surface of the RBD indicates that hydrophobic interactions may play a pivotal role when an antibody reaches the binding surface of the RBD, as many of these hydrophobic residues are directly involved in ACE2 recognition. This can be supported by some works of VNARs targeting SARS-CoV-2 RBD, which demonstrates that the binding of VNAR to the RBD in a strong affinity manner is mostly resourced from the establishment of hydrophobic interactions. The VNAR-2C02 reported by Ubah et al. (34) with high neutralizing activity binds to the RBD of SARS-CoV-2 relying heavily on the hydrophobic residues separated between the HV2 and CDR3 regions of the VNAR. Those residues were found interacting with the hydrophobic residues Ala348, Ala352, Leu452, Ile468, Phe490, and Leu492, located at the RBD binding surface. The VNAR-3B4, another potent SARS-CoV-2 neutralizer reported by Ubah et al., showed binding to the RBD distal to the ACE2 binding surface, which relied heavily on hydrogen bonding (34). A comparable dynamic was displayed by VNAR 20G6 described by Feng et al. (38) with an interaction mainly supported by hydrophobic interactions established from motifs in the CDR3 loop (38). Interestingly, VNAR 20G6 also binds to epitopes distal from ACE2 binding interface and maintained a good neutralizing effect. Similar results were observed by Valdovinos-Navarro et al. (40). Different sections of the CDR3, HV2, and CDR1 loops composed of hydrophobic residues appear to be pivotal when the VNAR SP240 approaches the RBD, as they interact with a hydrophobic patch formed from residues 445 to 498 in the RBD. In addition, the VNAR ShAb02 reported by Chen et al. (41) has a common mechanism with VNAR SP240 to approach the SARS-CoV-2 RBD. VNAR ShAb02 establishes multiple interactions with the RBD by also making use of the CDR3, HV2, and CDR1 loop. Moreover, they seemed to share epitopes located in the regions 346–356 and 445–453 of the RBD. This suggests a common neutralization mechanism among VNARs against SARS-CoV-2, which allows them to be grouped into class-II antibodies according to the classification of Barnes et al. (63). This class includes antibodies displaying a neutralization mechanism that involves direct competition with the ACE2 receptor. Our analysis suggests that this appears to be a common neutralization mechanism for SARS-CoV-2 in VNARs and is mainly driven by the hydrophobic portions distributed in the VNAR binding loops. This feature makes the interacting residues of the VNAR partly independent, to the extent that the residue identity on the RBD interface maintains its non-polar features. This partial independence could help to maintain a broad neutralizing spectrum and tackle emerging SARS-CoV-2 variants.

In addition, we found that none of the COVID-19 neutralizing VNARs reported to date have been humanized. The use of molecules of non-human origin for human treatments often raises concerns about their potential immunogenicity, as previously seen with the administration of murine mAbs in humans (64, 65). To enhance the use of non-human immunoglobulin scaffolds for therapeutic applications, additional steps of humanizing these molecules are required to minimize immunogenicity. The unique structural characteristics of VNARs could propose them as low immunogenic molecules, due to their small size, which results in a lower number of epitopes with immunogenic potential or to their rapid clearance from the blood. However, the concern about the potential immunogenicity of VNARs for therapeutic use in humans continues because of its divergent evolutionary origin and its low sequence identity (30%) to human immunoglobulin VH and VL domains sequences (24). To address this concern, a humanization strategy for VNARs has been reported (24), which largely maintains the antigen-binding specificity and affinity of the parental VNAR (66). We believe that, as a future perspective for these promising SARS-CoV-2 neutralizing VNAR molecules, the next step could address the humanization of their scaffolds, with the aim of reducing the potential immunogenicity that they may represent when administered to humans. In the particular case of SARS-CoV-2 neutralizing antibodies, Gauhar et al. (35) found an advantage in the non-human origin of VNARs. This is based on the rapid mutation rate of the SARS-CoV-2 that results in the appearance of new variants involving the selective mechanisms of the human host, where new emerging variants will be naturally selected for their ability to reinfect the human population and escape of their immune response. In this scenario, human antibodies could increase the selection pressure and the appearance of new variants with the potential to escape the immune response. Gauhar et al. (35) propose that, by not being part of the human immune response, VNARs would have a greater chance of retaining their neutralizing activity against emerging variant under selection pressure from Ig-based antibodies. However, more studies are needed to validate this hypothesis and to robustly elucidate the inhibition mechanism.

VNARs are a rich source of innovative therapeutic and diagnostic tools with broad intellectual property protection potential, demonstrated by ~3,020 submitted patents around the World. In particular, VNARs targeting SARS-CoV-2 have already been issued patents, and, currently, there are four different patent families on this subject, which were filed between September 2020 and November 2021. The work presented by Gauhar et al. (35) has led to the successful issuance of the premier patent pertaining to SARS-CoV-2 neutralizing VNARs, initially filed and granted within the United States (37) and subsequently pursued on the international stage through the Patent Cooperation Treaty (PCT), under reference WO2022/260877 (67). Currently, three additional patent families on VNARs against SARS-CoV-2 are undergoing the rigorous process of international evaluation through the PCT framework. Of these, two were first submitted in the United States, WO2022/060900 (68) derived from the work reported by Chen et al. (43) and WO2023/076881 (69), which has a direct connection to the earlier research detailed by Buffington et al. (44). Lastly, WO2023/CN077287 (70) was first submitted in China.

In conclusion, shark variable single domains represent an important alternative for the development of neutralizing molecules for pathogens of current concern, such as SARS-CoV-2. These molecules can be engineered into different formats either to take advantage of their small size or to exploit their flexible paratopes that can recognize protein motifs inaccessible to conventional antibodies. In addition, having synthetic antibody libraries, with high diversities, in which antibodies are found basically against any antigen, represented an important possibility for research groups to search for neutralizing antibodies against new and rapidly spreading pathogens such as SARS-CoV-2.

## Author contributions

OC-B: Conceptualization, Investigation, Supervision, Visualization, Writing – original draft, Writing – review & editing. BV-N: Investigation, Writing – original draft, Writing – review & editing. KC-L: Writing – original draft. NS-C: Investigation, Writing – original draft. AL-N: Conceptualization, Supervision, Writing – review & editing.
